# Two-Dimensional PtS_2_/MoTe_2_ van der Waals Heterostructure: An Efficient Potential Photocatalyst for Water Splitting

**DOI:** 10.3389/fchem.2022.847319

**Published:** 2022-02-14

**Authors:** Changqing Shao, Kai Ren, Zhaoming Huang, Jingjiang Yang, Zhen Cui

**Affiliations:** ^1^ School of Applied Engineering, Zhejiang Institute of Economics and Trade, Hangzhou, China; ^2^ School of Mechanical and Electronic Engineering, Nanjing Forestry University, Nanjing, China; ^3^ School of Mechanical Engineering, Wanjiang University of Technology, Ma’anshan, China; ^4^ School of Geely Automobile, Hangzhou Vocational and Technical College, Hangzhou, China; ^5^ School of Automation and Information Engineering, Xi’an University of Technology, Xi’an, China

**Keywords:** two-dimensional, heterostructure, photocatalyst, type-II band structure, water splitting

## Abstract

Recently, the energy shortage has become increasingly prominent, and hydrogen (H_2_) energy has attracted extensive attention as a clean resource. Two-dimensional (2D) materials show excellent physical and chemical properties, which demonstrates considerable advantages in the application of photocatalysis compared with traditional materials. In this investigation, based on first-principles methods, 2D PtS_2_ and MoTe_2_ are selected to combine a heterostructure using van der Waals (vdW) forces, which suggests a type-II band structure to prevent the recombination of the photogenerated charges. Then, the calculated band edge positions reveal the decent ability to develop the redox reaction for water splitting at pH 0. Besides, the potential drop between the PtS_2_/MoTe_2_ vdW heterostructure interface also can separate the photogenerated electrons and holes induced by the charge density difference of the PtS_2_ and MoTe_2_ layers. Moreover, the fantastic optical performances of the PtS_2_/MoTe_2_ vdW heterostructure further explain the promising advanced usage for photocatalytic decomposition of water.

## Introduction

Energy shortage and environmental problems have been widely concerning, which also urges new generation of green and efficient resources. Hydrogen (H_2_) has always been considered as a renewable and clean energy because of the environmentally friendly combustion product, H_2_O (Hernández-Alonso et al., 2009). Tremendous efforts have been explored to develop H_2_ (Ni et al., 2007; Carmo et al., 2013; Dincer and Acar, 2015), and the photocatalytic decomposition of water is very popular (Moniz et al., 2015), after the investigation the TiO_2_ was used as an electrode for splitting water via desirable light and temperature proposed by Fujishima and Honda (1972).

When the semiconductor acts as photocatalyst, the hydrogen evolution reaction (HER) can be induced by the higher potential of conduction band minimum (CBM) than −4.44 eV, while the lower potential of valence band maximum (VBM) than −5.67 eV can develop the oxygen evolution reaction (OER) (Wang et al., 2018a). Recently, two-dimensional (2D) materials have attracted abundant focus because of the discovery of fantastic physical and chemical performances (Geim and Novoselov, 2007; Sun et al., 2019, 2021; Ren et al., 2021a; Sun and Schwingenschlögl, 2021), which suggests advanced applications, such as photovoltaic (Long et al., 2016) and photocatalytic (Peng et al., 2018) devices, transistors (Tan et al., 2016), solar cells (Tsai et al., 2014), batteries (Sun and Schwingenschlögl, 2020) and thermoelectrics (Ren et al., 2020a), etc. Using 2D photocatalyst for water splitting is advantageous by the large specific surface area for the catalytic active site (Stoller et al., 2008). More importantly, the heterostructure with type-II band alignment can further provide prolonged lifetime of the photogenerated charges (Wang et al., 2014, 2020a, 2020b). Therefore, the investigations of nanostructured heterostructures are conducted such as boron nitride/cadmium sulfide (Wang et al., 2020c), CdO/arsenene (Ren et al., 2021b), ZnO/GeC (Wang et al., 2020d), transition metal dichalcogenides (TMDs)/BP (Ren et al., 2019), etc. Besides, type-I heterostructures also show considerable optical performances as photocatalysts (Ren et al., 2021c, 2021d; Zhu et al., 2021). Recently, TMD materials are widely studied because of their intriguing electronic (Shen et al., 2022), thermal (Ren et al., 2022), and optical (Luo et al., 2019) properties. The TMD materials also can be prepared by chemical vapor deposition (CVD) growth method (Wang et al., 2015; Tan et al., 2016). Especially, PtS_2_ monolayer has been synthesized by CVD (Zhao et al., 2019) and investigated to possess potential application as Z-scheme photocatalyst when stacking with the arsenene (Ren et al., 2020b) for water splitting. Furthermore, another TMD, MoTe_2_, has also been prepared by magnetron co-sputtering, and the Seebeck coefficient was obtained by ×2.89 10^4^ S/m (Shi et al., 2017). Besides, as a semiconductor (Conan et al., 1984), the monolayered MoTe_2_ shows tunable mobility (Qu et al., 2017). Therefore, both PtS_2_ and MoTe_2_ monolayers have promising electronic nature as a heterostructure photocatalyst together with the same hexagonal structure.

In this research, performing first-principles simulations, the electronic characteristic of the PtS_2_/MoTe_2_ heterostructure is investigated by a type-II band structure. Then, the photocatalytic mechanism is addressed by such decent band structure and band edge positions for water splitting. The potential drop and the charge density of the PtS_2_/MoTe_2_ heterostructure interface are also calculated. Finally, the optical performances of the monolayered PtS_2_, MoTe_2_, and PtS_2_/MoTe_2_ heterostructure are investigated.

### Computational Methods

In this investigation, we used the Vienna *ab initio* simulation package (VASP) to explore the first-principles calculation by the density functional theory (DFT) (Kresse and Furthmüller, 1996; Capelle, 2006). The projector augmented wave potential (PAW) (Kresse and Joubert, 1999) was used by generalized gradient approximation (GGA) (Perdew et al., 1996) and the Perdew–Burke–Ernzerhof (PBE) method was also considered in this work. The DFT-D3 function was conducted for the weak dispersion forces. To obtain the more real electronic and optical properties of the materials in the work, the Heyd–Scuseria–Ernzerhof hybrid method was employed (Heyd et al., 2005). Furthermore, the energy cut-off and the Monkhorst–Pack *k*-point grids were obtained by 500 eV and 15 × 15 × 1, respectively. To eliminate atomic interference between adjacent layers, vacuum thickness was set as 25 Å. Besides, the convergences were implemented by the force within 0.01 eV Å^−1^ and the energy limited in 0.01 meV. The binding energy (*E*_B_) was calculated using:

$$E_{B}=E\left(PtS_{2}/MoTe_{2}\right)-E\left(PtS_{2}\right)-E\left(MoTe_{2}\right),$$ (1)

where *E*(PtS_2_/MoTe_2_), *E*(PtS_2_), and *E*(MoTe_2_) represent the energy of the PtS_2_/MoTe_2_ system, monolayered PtS_2_, and MoTe_2_, respectively. The charge difference between the PtS_2_/MoTe_2_ interface is obtained by:

$$\Delta \rho =\rho \left(PtS_{2}/MoTe_{2}\right)-\rho \left(PtS_{2}\right)-\rho \left(MoTe_{2}\right),$$ (2)

where *ρ*(PtS_2_/MoTe_2_), *ρ*(PtS_2_) and *ρ*(MoTe_2_) are total charge density of the PtS_2_/MoTe_2_ heterostructure, primitive PtS_2_, and MoTe_2_ monolayers, respectively. The light absorption spectrum of the studied materials in this work is decided by:

$$\alpha \left(\omega \right)=\frac{\sqrt{2}\omega }{c}{\left\{{\left[\varepsilon_{1}^{2}\left(\omega \right)+\varepsilon_{2}^{2}\left(\omega \right)\right]}^{1/2}-\varepsilon_{1}\left(\omega \right)\right\}}^{1/2},$$ (3)

where $\varepsilon_{1}\left(\omega \right)$ and $\varepsilon_{2}\left(\omega \right)$ represent the dielectric constant for real and imaginary parameters, respectively. The speed of light, absorption coefficient, and the angular frequency are described by *c*, *α*, and *ω*, respectively.

## Results and Discussion

**FIGURE 1:**
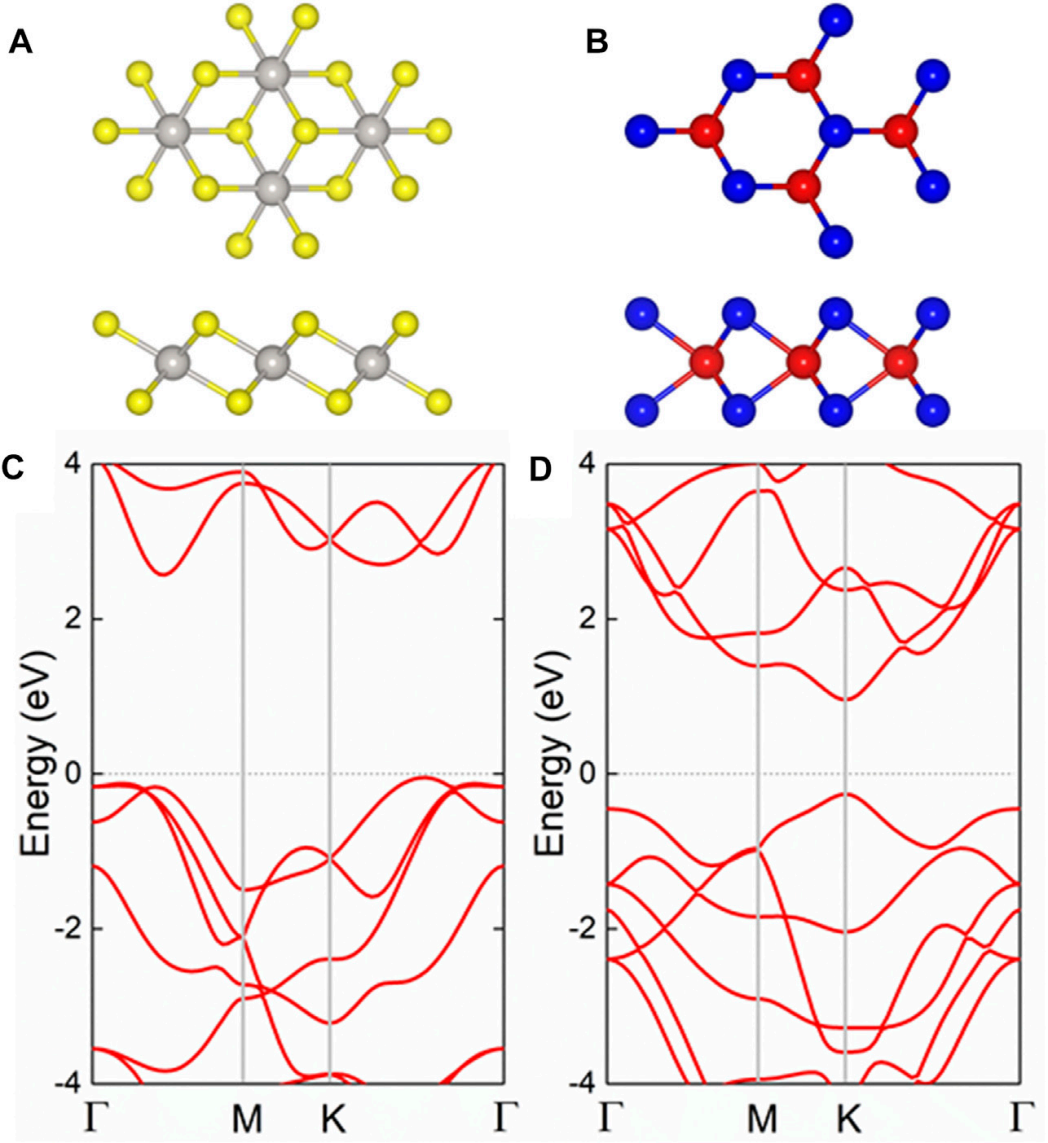
The **(A,B)** geometric and **(C,D)** band structures of the pristine **(A,C)** PtS_2_ and **(B,D)** MoTe_2_ monolayers; the yellow, gray, red, and blue balls represent S, Pt, Mo, and Te atoms, respectively; the Fermi level is expressed as 0 using gray dash line.

The PtS_2_ and MoTe_2_ monolayers possess hexagonal honeycomb structure, shown in Figures 1A,B, respectively. And the structures of the PtS_2_ and MoTe_2_ monolayers are optimized, first, by the lattice parameters of 3.564 and 3.529 Å, respectively. Besides, the band structure of the PtS_2_ and MoTe_2_ monolayers are also calculated by HSE06 method, demonstrated in Figures 1C,D, respectively, suggesting both layered materials are semiconductors. The PtS_2_ monolayer possesses an indirect bandgap of 2.60 eV with the CBM and VBM located between the Γ and M points. Furthermore, the MoTe_2_ monolayer has a direct bandgap calculated to be 1.22 eV by the CBM and VBM at K point. The obtained lattice parameters and bandgaps of the monolayered PtS_2_ and MoTe_2_ are in good agreement with other investigations (Nguyen et al., 2019; Wang et al., 2021). Besides, the optimized bond length of the Pt−S and Mo−Te are 2.40 and 2.74 Å, respectively.

**FIGURE 2:**
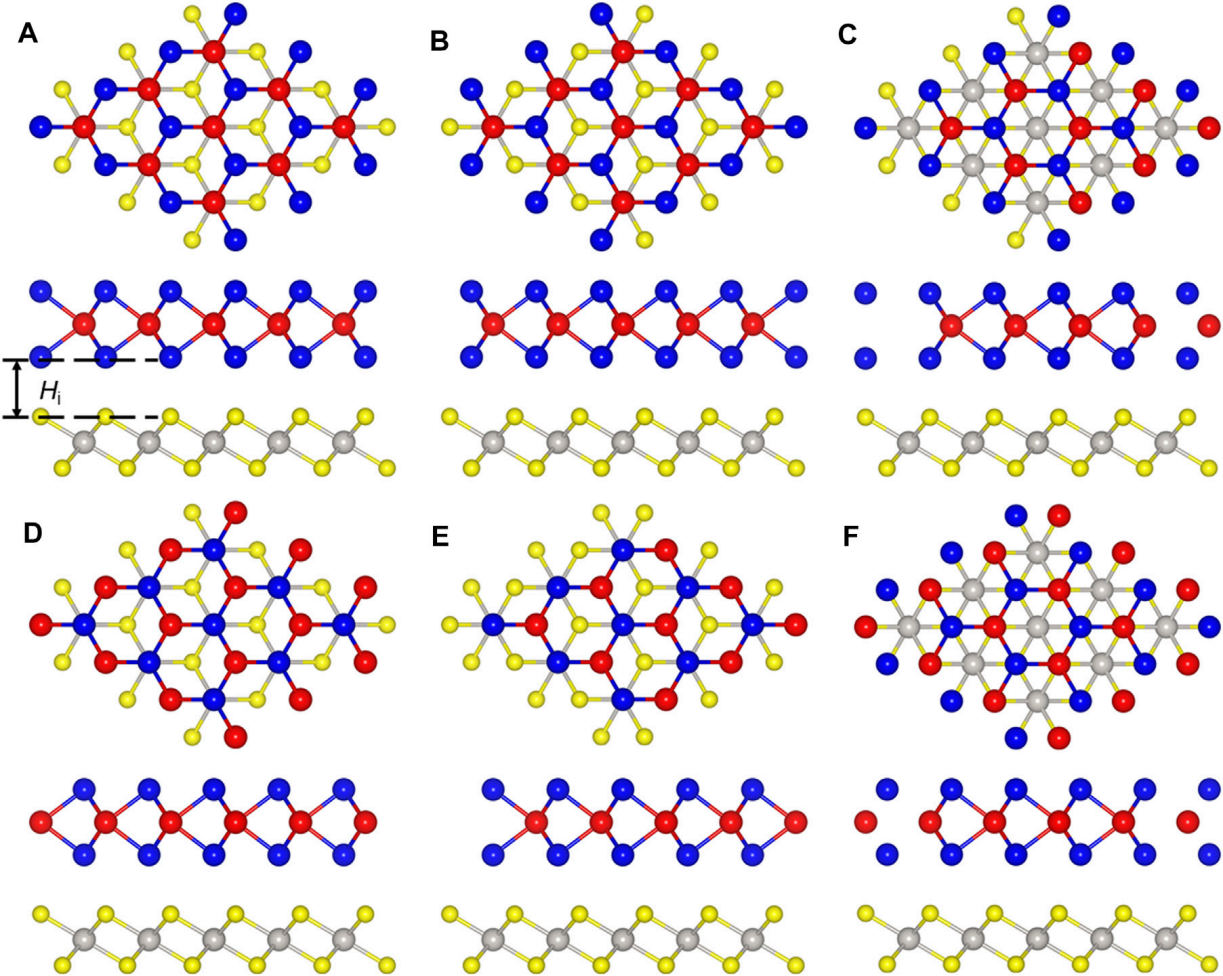
The stacking styles of the PtS_2_/MoTe_2_ heterostructure constructed by **(A)** PM-1, **(B)** PM-2, **(C)** PM-3, **(D)** PM-4, **(E)** PM-5, and **(F)** PM-6, respectively.

The PtS_2_/MoTe_2_ heterostructure can be constructed by six different configurations considering the high symmetry, named PM-1, PM-2, PM-3, PM-4, PM-5, and PM-6 styles. To decide the most stable staking structure, the binding energy of these different configurations are calculated, and the lowest binding energy is about −28.10 meV Å^−2^ for PM-6 stacking style, suggesting the van der Waals (vdW) forces between the interface of the PtS_2_/MoTe_2_ heterostructure (Chen et al., 2013). The obtained bond length of the Pt−S and Mo−Te in the PtS_2_/MoTe_2_ heterostructure are 2.39 and 2.73 Å, which is almost the same as that of the original single-layer material, further demonstrating the vdW interaction. Moreover, the interlayer height (*H*_i_) shown in Figure 2A of the PtS_2_/MoTe_2_ vdW heterostructure with PM-6 stacking style is calculated by 2.87 Å. Besides, the following obtained works are based on such PM-6 stacking style.

**FIGURE 3:**
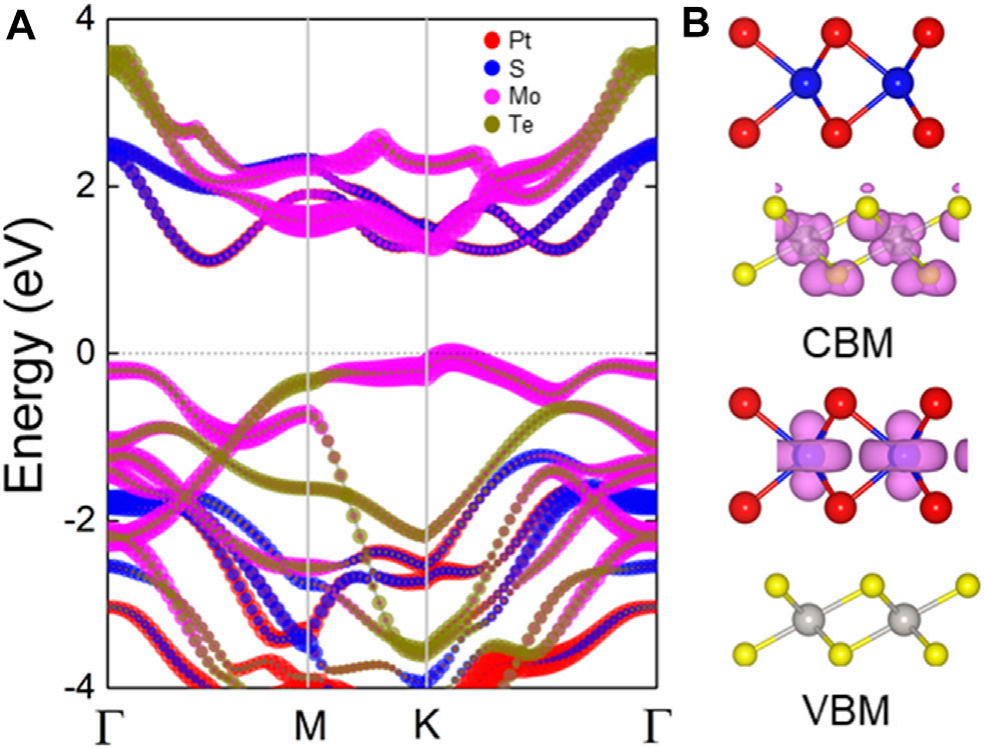
**(A)** The HSE method obtained projected band structure and the **(B)** band-resolved charge densities of the PtS_2_/MoTe_2_ vdW heterostructure; the Fermi level is expressed as 0 using gray dash line.

The projected band structure of the PtS_2_/MoTe_2_ vdW heterostructure are calculated in Figure 3A, which shows that the CBM and the VBM of the heterostructure are contributed by the PtS_2_ and MoTe_2_ monolayers, respectively, suggesting an intrinsic type-II band structure. One can see that the PtS_2_/MoTe_2_ vdW heterostructure also is a semiconductor by an indirect bandgap of 1.26 eV that the CBM is located between the Γ and M points, while the CBM exists at K point. Besides, the obtained band-resolved charge densities, explained by Figure 3B, of the PtS_2_/MoTe_2_ vdW heterostructure can further demonstrate the different layered contribution to CBM and VBM.

**FIGURE 4:**
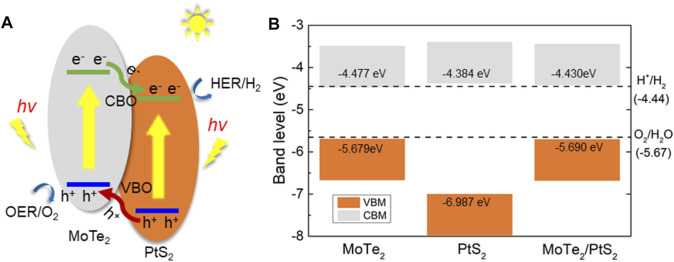
**(A)** The photogenerated charges migration path and **(B)** the band alignment of the PtS_2_/MoTe_2_ vdW heterostructure.

The type-II band structure of the PtS_2_/MoTe_2_ vdW heterostructure can provide the ability to separate the photogenerated electrons (PE) and the holes used as a photocatalyst for water splitting. As shown in Figure 4A, the PtS_2_/MoTe_2_ vdW heterostructure takes in the energy of the photon larger than the bandgap of the PtS_2_ and MoTe_2_ layers; the PE are excited by the CB of the PtS_2_ and MoTe_2_ layers, and thus, the photogenerated holes (PH) stay at the VB at the same time. Then, the PE at the CB of the MoTe_2_ layer will move to the CB of the PtS_2_ layer because of the promoting of the conduction band offset, named CBO in Figure 4A. Similarly, the PH at the PtS_2_ layer also can transfer to the VB of the MoTe_2_ layer by the development of the valence band offset, denoted by VBO in Figure 4A. Therefore, the PEs are continuously promoted from the CB of the MoTe_2_ layer to PtS_2_ layer, while the PHs keep moving from the VB of the PtS_2_ layer to the MoTe_2_ layer under continuous solar photodynamic, which induces a PE and PH circulating flow (Wang et al., 2018b).

Furthermore, the band edge positions of the PtS_2_/MoTe_2_ vdW heterostructure is also calculated in Figure 4B to investigate the photocatalytic driving potential for water splitting. At pH 0, the standard potential energy of the HER and the OER are −4.44 and −5.67 eV, respectively (Wang et al., 2018a). The obtained band alignment of the monolayered PtS_2_, MoTe_2_, and the PtS_2_/MoTe_2_ vdW heterostructure is demonstrated by Figure 4B, which shows that the monolayered PtS_2_ and the PtS_2_/MoTe_2_ vdW heterostructure have suitable band edge positions to induce the HER and OER at pH 0. However, the PtS_2_ cannot separate the PE and PH compared with the type-II band structure in the PtS_2_/MoTe_2_ vdW heterostructure. Thus, the PtS_2_/MoTe_2_ vdW heterostructure can be considered as a potential photocatalyst to decompose the water.

**FIGURE 5:**
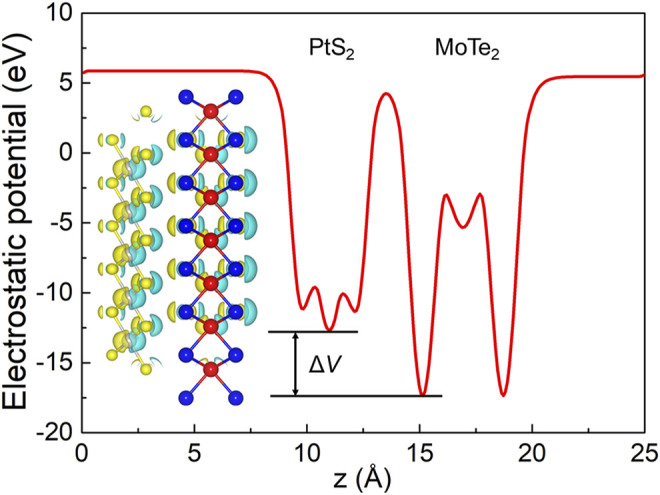
The potential drop between the interface of the PtS_2_/MoTe_2_ vdW heterostructure; the inset represents the charge density. The isosurface lever was set at 0.015|e|.

The interfacial performances of the PtS_2_/MoTe_2_ vdW heterostructure are assessed by charge density difference (Δ*ρ*) and the potential. The charge density difference is calculated by Bader charge analysis (Tang et al., 2009; Henkelman et al., 2006), shown in the inset of Figure 5; the cyan and yellow marks denote the taking and giving of electrons, suggesting that the PtS_2_ and MoTe_2_ monolayers act as receivers and donors, respectively. Besides, the obtained charge transfer between the PtS_2_ and MoTe_2_ vdW heterostructure is 0.047 electrons. Furthermore, such charge transfer also can induce a potential drop (Δ*V*) across the PtS_2_/MoTe_2_ vdW heterostructure interface, explained by Figure 5. From the PtS_2_ layer to the MoTe_2_ layer, the potential decreases by 4.672 eV, which is higher than that in arsenene/GaS (4.215 eV) (Li et al., 2021), AlN/Zr_2_CO_2_ (0.663 eV) (Ren et al., 2021c), and Hf_2_CO_2_/GaN (3.752 eV) (Ren et al., 2021d) heterostructures. It is worth noting that the potential drop also can provide decent assistance in the process of the separation of photogenerated charges (Wang et al., 2018b).

**FIGURE 6:**
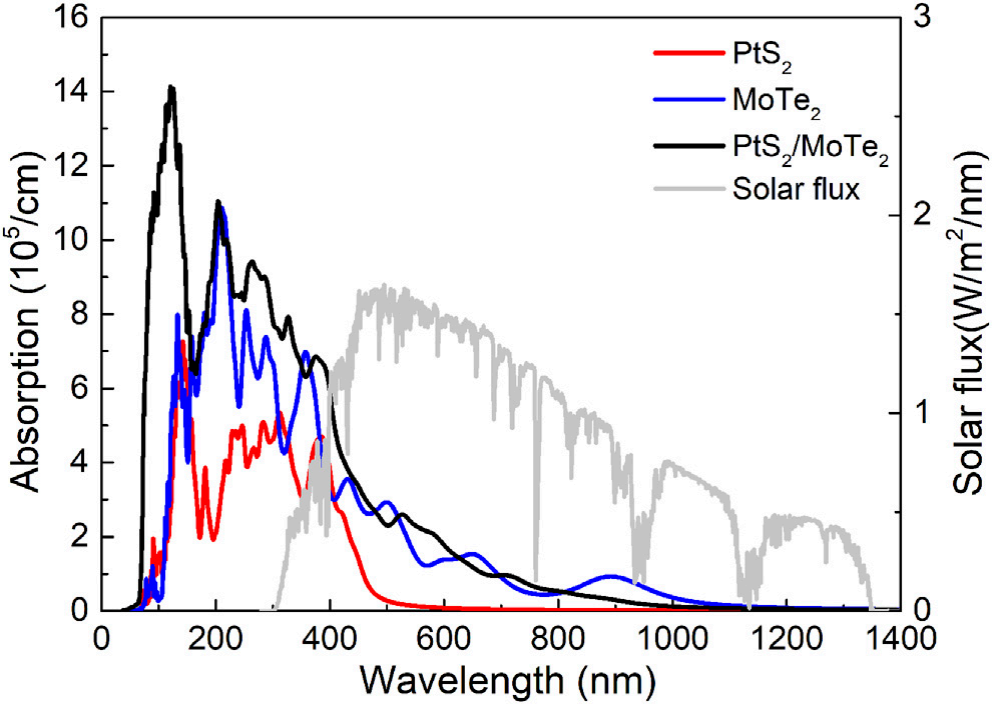
The calculated optical absorption spectrum of the monolayered PtS_2_, MoTe_2_, and PtS_2_/MoTe_2_ vdW heterostructure.

Used as a photocatalyst for water splitting, light absorption capacity also has a vital role. The light absorption properties of the monolayered PtS_2_, MoTe_2_, and the PtS_2_/MoTe_2_ vdW heterostructure are evaluated and shown in Figure 6. The PtS_2_/MoTe_2_ vdW heterostructure obviously can improve the optical ability of the monolayered PtS_2_, MoTe_2_ in ultraviolet and visible regions. In the visible wavelength range, the absorption peaks of the PtS_2_ and MoTe_2_ monolayers and the PtS_2_/MoTe_2_ vdW heterostructure are obtained at 4.70 × 10^5^, 2.90 × 10^5^, and 2.57 × 10^5^ cm^−1^ with wavelengths of 384, 505, and 531 nm, respectively. It is worth noting that MoTe_2_ monolayer and the tS_2_/MoTe_2_ vdW heterostructure possess another absorption peak at 1.53 × 10^5^ and 6.82 × 10^5^ cm^−1^ with wavelengths of 650 and 380 nm, respectively. The results show that the PtS_2_ and MoTe_2_ monolayers and the PtS_2_/MoTe_2_ vdW heterostructure have excellent optical performances, which is higher than other reported 2D heterostructures, such as WSSe/Mg(OH)_2_ (4.295 × 10^5^ cm^−1^) (Lou et al., 2021), arsenene/GaSe (5.868 × 10^5^ cm^−1^) (Li et al., 2021), etc.

## Conclusions

Using DFT calculations, the structural and electronic nature of the monolayered PtS_2_ and MoTe_2_ are investigated as semiconductors. Then, the PtS_2_/MoTe_2_ heterostructure is constructed by vdW interactions, also showing a type-II band alignment to prevent the PE and PH from recombining. More importantly, the PtS_2_/MoTe_2_ vdW heterostructure possesses desirable band edge positions to boost the HER and OER in the PtS_2_ and MoTe_2_ layers, respectively. In the PtS_2_/MoTe_2_ vdW heterostructure, the PtS_2_ layer obtains 0.047 electrons from the MoTe_2_ layer, which induces a 4.672 eV potential drop. Furthermore, all these monolayered PtS_2_ and MoTe_2_ and the PtS_2_/MoTe_2_ vdW heterostructure show excellent optical properties; particularly, the PtS_2_/MoTe_2_ vdW heterostructure suggests a novel light absorption performance in the visible range, revealing the potential application such as new energy vehicle fuel cell photocatalyst.

## Data Availability

The raw data supporting the conclusions of this article will be made available by the authors, without undue reservation.
